# Dental Anxiety as a Potential Bottleneck in Oral–Systemic Health Pathways: A Conceptual Mapping Review of Review Articles

**DOI:** 10.3390/dj14040227

**Published:** 2026-04-10

**Authors:** Mika Kajita, Vesa Pohjola, Gerald Humphris, Satu Lahti

**Affiliations:** 1 Department of Community Dentistry, University of Turku, 20014 Turku, Finland; vesa.pohjola@utu.fi (V.P.); satu.lahti@utu.fi (S.L.); 2 Research Unit of Population Health, University of Oulu, 90014 Oulu, Finland; 3 School of Medicine, University of St Andrews, St Andrews KY16 9TF, UK; gmh4@st-andrews.ac.uk

**Keywords:** dental fear, behavioural bottleneck, oral–systemic link, public health, health equity

## Abstract

**Background/Objectives:** Although many studies have examined the determinants and management of dental anxiety (DA), its broader placement as a potential bottleneck along oral–systemic health pathways, from the determinants of DA to consequences through dental avoidance, oral outcomes, psychosocial impacts, and possible systemic health outcomes, has not been mapped across the review literature. This review aimed to conceptually map how existing DA reviews are distributed across this pathway, whether this broad framing changed across 5-year periods, and how systemic health outcomes were framed. **Methods:** We conducted a conceptual mapping review of DA-focused review articles published between 2005 and 2025. PubMed and Scopus were searched for English-language narrative, systematic, scoping and umbrella reviews and meta-analyses addressing the determinants or consequences of DA. One reviewer screened records, extracted review characteristics, and classified each review into predefined domains using binary framed/not framed coding rules. A structured AI-assisted prompt was used only to support full-text evaluation across domains; all final coding decisions were made by the reviewer. **Results:** The search identified 851 records; after removing 426 duplicates, 425 unique records were screened, and 39 reviews met the inclusion criteria. Framing concentrated on environmental and psychological determinants and on the pathway from DA to avoidance and poor oral health, whereas broader consequences, including shame, OHRQoL, and systemic health outcomes, were less consistently framed. Across 5-year periods, the broad pattern of framing remained relatively stable. Systemic health outcomes were framed in only a minority of reviews. **Conclusions:** Future research should test hypothesized pathways from DA to broader health consequences using clearly specified bridge mechanisms and appropriate temporal designs.

## 1. Introduction

Dental anxiety (DA) is common and is consistently associated with avoidance of dental care, delayed treatment, and poorer oral health [1,2], suggesting that it may contribute to broader patterns of oral health disadvantage and inequality. Current global policy increasingly frames oral health as part of overall health [3], while oral epidemiology has shown robust links between poor oral health and systemic conditions [4,5]. Within this broader context, DA can be understood as a behavioural barrier to dental attendance, with potential consequences that may extend beyond the mouth.

**Figure 1:**
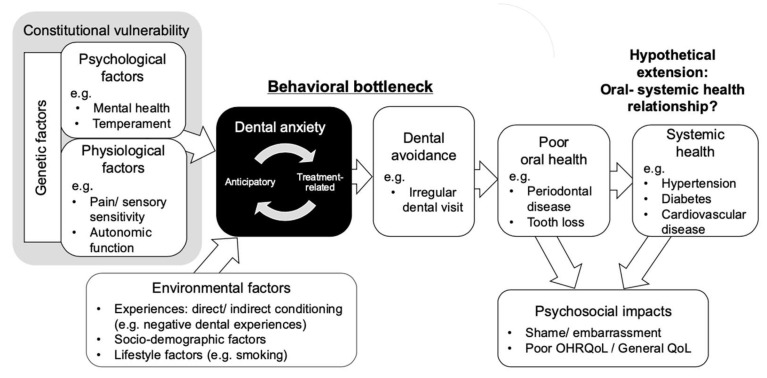
Hypothetical conceptual model of dental anxiety from determinants to consequences, used to define the domain framework for mapping. Abbreviations: OHRQoL, oral health-related quality of life; QoL, quality of life.

To operationalise this mapping, we draw on existing theoretical models of determinants and consequences of DA to define the conceptual pathway used in this review. Across the DA literature, conceptual models have consistently suggested that DA arises from interacting vulnerabilities and experiences, and that it has consequences through avoidance and care. Early learning-based models emphasised conditioning, vicarious learning, and negative information [6], whereas later models distinguished external dental experiences from internal vulnerability, including trait anxiety and related predispositions [7]. Subsequent frameworks further highlighted cognitive appraisals such as uncontrollability and unpredictability and integrated these strands into broader biopsychosocial accounts [8,9,10]. On the consequence side, negative reinforcement was proposed to maintain avoidance, thereby sustaining DA over time [11,12,13] and the vicious cycle model positioned avoidance as a key maintaining mechanism, linking DA to delayed attendance, oral deterioration, shame, and broader psychosocial burden [14]. Later extensions also incorporated broader psychosocial consequences, including reduced self-esteem, impaired social functioning, social withdrawal, and dental shame [15,16]. Taken together, these models do not merely describe DA; they provide the theoretical basis for a pathway extending from vulnerability and learning processes to avoidance, oral outcomes, and psychosocial consequences. In the present review, this theoretical tradition was used to define the working framework and coding domains shown in Figure 1.

At the same time, whether the DA literature has been synthesised in a way that reflects this broader pathway remains unclear. DA-specific conceptual models and DA-focused reviews have largely concentrated on psychological determinants, dental experiences, avoidance, and oral outcomes (Figure 1). By contrast, the oral–systemic literature has developed substantially outside the DA field, particularly through large bodies of work on periodontal disease and systemic diseases [4,5]. As a result, it remains uncertain how far review-level synthesis in DA has extended along the hypothesised pathway from determinants to avoidance, oral consequences, psychosocial impacts, and possible systemic implications. For the purposes of the present review, we therefore extended the integrated DA framework by adding systemic health outcomes as a hypothetical downstream domain.

The purpose of this study was to characterise how the review literature on DA frames DA within a broader pathway from determinants to consequences, including possible oral–systemic implications. Specifically, we examined where review articles cluster along this pathway, whether the overall pattern changed across 5-year periods, and whether and how systemic health outcomes were framed within it. This approach allowed us to assess both the growth of the literature and whether its broad conceptual framing remained stable or changed over time.

## 2. Materials and Methods

### 2.1. Design and Reporting

This study was conducted as a conceptual mapping review of review articles. Because no formally agreed reporting guideline for mapping reviews is yet available, reporting was primarily informed by the draft mapping review recommendations proposed by Li et al. (2025) [17]. The draft mapping review checklist is provided in Supplementary File S1. As the review also involved a structured search, explicit eligibility criteria, and record selection, we additionally provided detailed reporting of the search, screening, and study selection process to improve transparency in a manner informed by commonly expected evidence-synthesis reporting elements. The review was not designed or conducted as a scoping review and was therefore not reported in accordance with the Preferred Reporting Items for Systematic Reviews and Meta-Analyses extension for Scoping Reviews (PRISMA-ScR). This mapping review was not registered, and no a priori protocol was published. No stakeholders (e.g., patients, clinicians, or policymakers) were involved in formulating the review question, conducting the review, or interpreting the findings; rather, the review was initiated and conducted by the authors.

### 2.2. Eligibility Criteria

Articles were eligible for inclusion if they explicitly addressed DA, dental fear or phobia as a primary focus and discussed either determinants or consequences of DA and were published from 2005 to 2025. Articles were excluded if they did not treat DA as a central topic, focused exclusively on measurement properties or prevalence estimates without reference to determinants or consequences, focused solely on clinically oriented management or intervention approaches, or lacked an accessible abstract in English.

### 2.3. Search Strategy

To clarify how DA has been positioned within the existing literature, a structured database search of PubMed and Scopus was performed on 14 May 2025. Because our objective was to characterise the scope of DA-focused scholarship, we intentionally anchored the search to “dental anxiety/fear/phobia” and included only reviews that explicitly framed their topic in these terms (in the title/abstract/keywords), rather than broader reviews of dental attendance, avoidance, or access barriers that may not be DA-framed. In PubMed, the search string was: (“dental anxiety”[Title/Abstract] OR “dental fear”[Title/Abstract] OR “dental phobia”[Title/Abstract]) AND (review[Publication Type] OR “systematic review”[Title/Abstract] OR “narrative review”[Title/Abstract] OR “scoping review”[Title/Abstract] OR “meta-analysis”[Title/Abstract]) AND (“2005/01/01”[Date—Publication]: “2025/12/31”[Date—Publication]) AND (english[Language]). Searches were restricted to English-language publications. To widen coverage, Scopus was searched using the following query: TITLE-ABS-KEY(“dental anxiety” OR “dental fear” OR “dental phobia”) AND TITLE-ABS-KEY(“review” OR “systematic review” OR “narrative review” OR “scoping review” OR “meta-analysis”) AND PUBYEAR > 2004 AND PUBYEAR < 2026 AND (LIMIT-TO(DOCTYPE, “re”)) AND (LIMIT-TO(LANGUAGE, “English”)). The search was limited to English-language reviews published between 2005 and 2025. Reference lists of included articles were screened to identify any additional eligible reviews.

### 2.4. Study Selection and Data Extraction

All screening and data extraction were performed by a single reviewer (M.K.). Titles and abstracts were screened for relevance, with full texts assessed when necessary. Records were imported into Rayyan, a web-based screening tool for evidence synthesis projects, to support organised screening and reduce clerical error. For each included review, information was extracted on the review type (narrative, systematic, meta-analytic, scoping, or umbrella/overview) and on its substantive focus within the DA pathway. When reviews used mixed approaches, we classified them using a hierarchical rule (meta-analysis > systematic review > scoping > narrative) based on the methods contributing most directly to our mapping question. Each review was classified into one or more of the following domains: determinants (genetic, psychological, physiological, or environmental [experiences, sociodemographic factors and lifestyle factors]), dental avoidance or irregular attendance, oral health outcomes (such as dental caries or periodontal status), psychosocial impacts (shame/embarrassment, oral-health–related quality of life [OHRQoL], general quality of life [QoL]), and systemic health outcomes.

Coding rules: Coding was conducted by a single reviewer (M.K.) using a binary scheme (1 = framed; 0 = not framed). The operational definitions used for this classification are summarised in Table 1. In this review, “framed” referred to whether a domain played a meaningful role in how dental anxiety was positioned, explained, or contextualised within the review, rather than being noted only in passing. Future-oriented statements were coded as framed only when they were clearly integrated into that framing.

The full-text coding was supported by a structured AI-assisted prompt (OpenAI; GPT-5.4 Thinking, accessed in March 2026). AI was used to apply the coding rules systematically across all predefined domains to help identify relevant supporting passages in the source text. AI outputs were used only to generate provisional codes and supporting text locations. All outputs were checked against the full text by the reviewer (M.K.), and any reviewer changes to the AI-generated coding were logged together with the reason for the change. All final coding decisions were made by the reviewer. AI did not determine study inclusion, final domain coding, interpretation of findings, or the conclusions of the review.

For consequence domains (avoidance, oral outcomes, shame, OHRQoL, general QoL, and systemic outcomes), we applied a strict consequence-only rule: these were coded only when framed as consequences of dental anxiety (DA→X). If framed as antecedents/correlates (X→DA), the consequence domain was coded 0 and relevant content was captured under determinants (genetic, psychological, physiological, environmental) as appropriate. Procedure-related painful dental experiences were coded as environmental exposures, whereas stable pain/sensory sensitivity traits were coded as physiological determinants. The full prompt used for AI-assisted cross-checking and one example of the unedited response is provided in Supplementary File S2.

The reporting of generative AI use was guided by the Generative Artificial intelligence tools in MEdical Research (GAMER) Statement [18] and the completed GAMER checklist is provided in Supplementary File S3. No personal data, patient data, or confidential information were entered into the AI tool.

We did not conduct a formal quality appraisal (risk-of-bias assessment) of included reviews because our aim was to map what topics were covered and how they were framed in the review literature, rather than to synthesise effect sizes or make judgements about intervention effectiveness.

### 2.5. Data Summarisation and Mapping

No meta-analysis was attempted. Instead, we used a conceptual mapping approach to examine how review articles framed dental anxiety (DA) across predefined determinant and consequence domains. The aim was not to synthesise empirical effect sizes or the strength of associations, but to map how far DA was conceptually extended within the review literature, from upstream determinants to downstream consequences of avoidance, oral outcomes, psychosocial impacts, and hypothesised systemic health outcomes.

Review characteristics were first summarised descriptively, including the distribution of review types across 5-year periods. Included reviews were organised using a binary review-by-domain matrix (framed = 1, not framed = 0) across predefined determinant and consequence domains. We then summarised, for each 5-year period, the proportion of reviews in which each domain was coded as framed. These temporal patterns were presented as a domain heatmap. Next, overall framing frequencies across the full review set were integrated into a main mapping figure showing the proportion of reviews in which each domain was framed along the proposed conceptual pathway. Because systemic health outcomes were relatively infrequently framed and varied in how explicitly they were connected to DA, these reviews were additionally summarised in a separate descriptive table detailing how systemic health was framed and how specific that framing was.

The main mapping figure was created manually in Microsoft PowerPoint, and the domain heatmap was generated in Microsoft Excel from the coded review-by-domain matrix. Although artificial intelligence was used only to support provisional coding, all mapping outputs and visualisations were produced and finalised manually by the reviewer.

We did not pre-define quantitative thresholds for “adequate evidence,” because this review did not aim to evaluate evidentiary strength. Instead, we interpreted frequently framed domains as areas of conceptual concentration in the review literature, and infrequently framed domains as areas that remained conceptually less developed or less consistently integrated

## 3. Results

**Figure 2:**
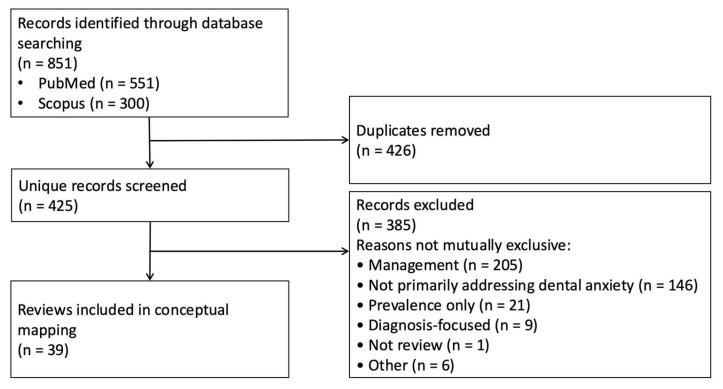
Record selection flow for the conceptual mapping review.

In this conceptual mapping review of review articles published between 2005 and 2025, searches in PubMed (*n* = 551) and Scopus (*n* = 300) yielded a total of 851 records. After removal of 426 duplicates, 425 unique records were screened, and 39 reviews met the inclusion criteria for the conceptual mapping review (Figure 2). Detailed counts for exclusions at the title/abstract and full-text stages were not retained separately; therefore, the figure summarises the overall record selection process rather than a PRISMA-style stage-specific flow diagram.

The full list of included reviews (*n* = 39) is provided in Table S1. Table S1 also includes the provisional AI-generated domain codes and, where the reviewer assigned a different final code, the recorded reason for that change. Across 390 article–domain decisions (10 domains × 39 reviews), such changes were made in 7 instances. The list of all screened records is publicly available in an open repository at https://doi.org/10.5281/zenodo.18523631, accessed on 8 February 2026.

Among the 39 included reviews, the most common format was narrative reviews (19/39, 48.7%), followed by systematic reviews (12/39, 30.8%) and meta-analyses (7/39, 17.9%), and umbrella reviews were rare (1/39, 2.6%).

**Figure 3:**
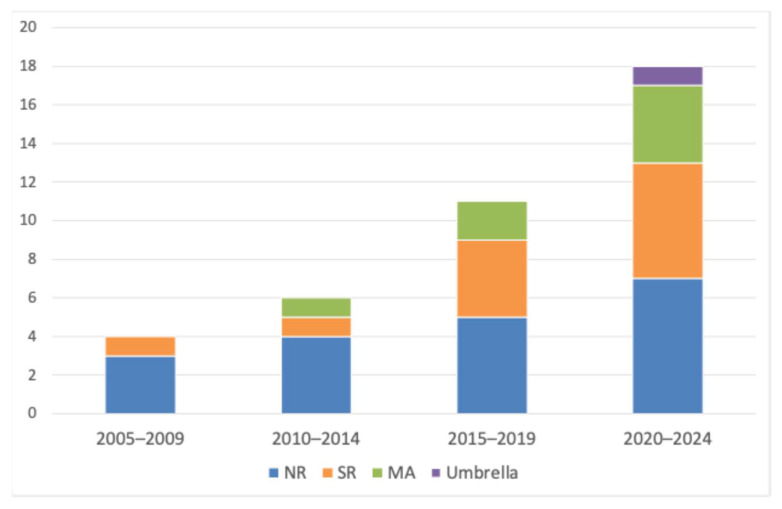
Temporal evolution of review types in the dental anxiety review literature (2005–2025). No included reviews were published in 2025; therefore, the final period shown is 2020–2024. Counts represent included review articles in this mapping review, categorised by publication period and primary review type (narrative review, systematic review, meta-analysis, umbrella review). The figure illustrates the growth and diversification of synthesis approaches within the dental anxiety review literature.

The number of review articles on DA increased over time, particularly after 2015. Earlier periods were dominated by narrative reviews, whereas later periods included a larger number of systematic and meta-analytic reviews, indicating increasing methodological diversity in the synthesis of the literature (Figure 3). The search covered publications from 1 January 2005 to 31 December 2025. However, no included reviews were published in 2025; therefore, temporal analyses were presented across four complete 5-year periods (2005–2009, 2010–2014, 2015–2019, and 2020–2024).

**Figure 4:**
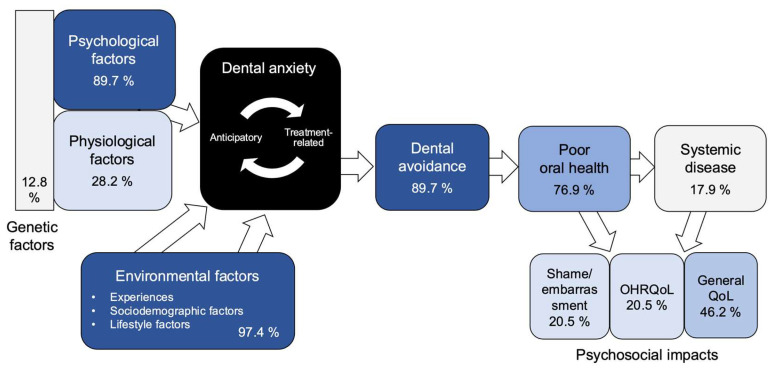
Mapping results showing the proportion of included review articles in which each domain was framed along the conceptual pathway. Percentages indicate the proportion of included reviews (*n* = 39) in which each domain was framed. Darker shading indicates a higher proportion. Abbreviations: OHRQoL, oral health-related quality of life; QoL, quality of life.

Figure 4 maps the proportions of reviews in which each domain was framed onto the conceptual pathway used for classification. The figure shows that review-level framing is most concentrated on environmental and psychological determinants and on the pathway from dental anxiety to avoidance and poor oral health. By contrast, broader downstream extensions are less consistently framed, particularly for shame, OHRQoL, and systemic health outcomes, while general quality of life occupies an intermediate position.

**Figure 5:**
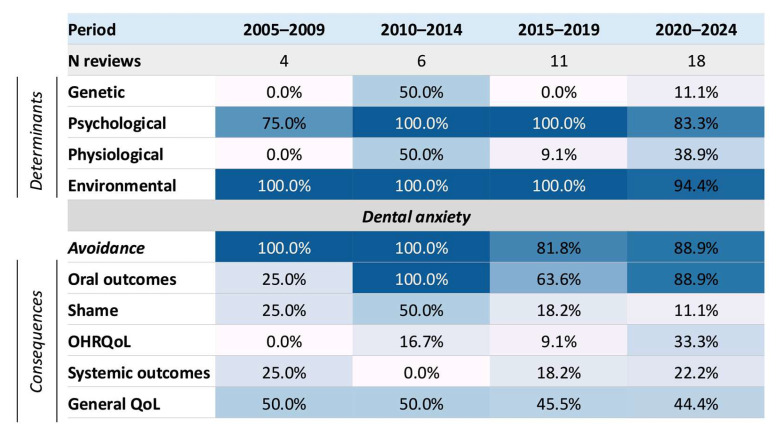
5-year trends in domain framing across review articles. Values show the percentage of reviews in each 5-year period in which the domain was framed. No included reviews were published in 2025; therefore, the final period shown is 2020–2024. Avoidance is shown as the central behavioural pathway linking dental anxiety to later consequences. Abbreviations: OHRQoL, oral health-related quality of life; QoL, quality of life.

Figure 5 shows a 5-year domain trend heatmap. The heatmap suggests that, despite growth in the number of review articles, the broad pattern of domain framing remained relatively stable across 5-year periods. Review-level framing consistently concentrated on psychological and environmental determinants and on the core pathway from dental anxiety to avoidance and oral outcomes, whereas broader psychosocial and systemic consequence domains remained less consistently framed.

We additionally examined the seven reviews in which systemic health outcomes were framed and summarised the context and specificity of this framing in Supplementary Table S2. Among the seven reviews that framed systemic health outcomes, three used only broad systemic-health wording, three provided moderate framing with at least one named disease example or tentative downstream pathway, and only one integrated systemic outcomes more explicitly into the main review framing.

## 4. Discussion

This conceptual mapping review was designed to show how far DA-framed review literature extends beyond dental anxiety itself across a broader conceptual pathway. Rather than synthesising effect sizes or levels of evidence, it aimed to make the conceptual distribution of framed domains visible across the review literature. The findings should therefore be interpreted as review-level patterns of framing, not as equivalent evidence synthesis across review types. This review showed that the DA review literature has grown in both number and methodological diversity over time, particularly after 2015. However, the broad pattern of review-level framing remained relatively stable across periods. Reviews consistently concentrated on psychological and environmental determinants and on the core pathway from DA to avoidance and poor oral health. By contrast, broader consequence domains, including shame, OHRQoL, and systemic health outcomes, were framed less consistently, and systemic health was usually framed only in broad or tentative terms.

Reviews consistently concentrated on psychological and environmental determinants and on the core pathway from DA to avoidance and poor oral health. One possible interpretation of this relative stability is that the DA review literature continues to be organised around a well-established framework linking these domains. This likely reflects the enduring usefulness of classical conditioning [6] theory and vicious-cycle models [14], which have helped clarify both the maintenance of DA and its clinical significance. The relatively stable pattern seen in our heatmap may therefore reflect the persistence of a practical framework rather than a simple lack of change. At the same time, from a public health perspective, this concentration may suggest that some dimensions of DA remain less fully developed in review-level framing. In particular, greater attention to genetic and biological vulnerability, as well as to broader psychosocial and systemic consequences, may help place DA more clearly within wider pathways of oral and general health disadvantage.

Across the seven reviews that framed systemic health outcomes, integration was usually limited: three used only broad systemic-health wording, three provided moderate framing with at least one named disease example or a tentative broader pathway, and only one integrated systemic outcomes more explicitly into the main review framing. Beaudette et al. (2017) could extend the pathway to systemic health because they adopted an interdisciplinary framework linking oral health, dietary choices, and chronic disease risk, with DA positioned as an upstream driver [19]. This suggests that approaches that include bridge mechanisms such as diet and nutrition, chronic inflammation, and lifestyle factors may be needed to capture broader consequences beyond oral health. More tentative examples were seen in Aburas et al. [20] and Ying et al. [21], which named specific diseases but treated systemic outcomes more peripherally. Ying et al. most clearly described the gap, noting that while children’s dental fear is consistently linked to poor oral and mental health, whether DA predicts later systemic complications has not been directly studied [21]. Thus, extension to systemic outcomes remains plausible but under-tested and should be treated as a hypothesis rather than established causal evidence. Taken together, clarifying the public health impact of DA will require empirical studies, ideally longitudinal, that treat systemic disease as an outcome and test pathways involving bridge mechanisms.

Links between oral diseases and major systemic conditions such as diabetes, cardiovascular disease, and adverse pregnancy outcomes are now well established. Evidence from prospective cohorts and meta-analyses supports the role of chronic oral infection and inflammation in systemic disease pathways [4,22,23,24]. This scientific consensus has influenced global health policy. The World Health Organization now includes oral health within the framework of Universal Health Coverage and emphasises the importance of removing barriers that limit equitable access to oral health care [3]. Financial and organisational barriers are widely recognised, but DA also acts as a major behavioural barrier to dental attendance [25,26,27]. Viewing DA only as an individual psychological concern or a clinical management issue may therefore be too narrow. A public health perspective highlights DA as a potential bottleneck that affects utilisation, prevention, and timely treatment, and may help to clarify how avoidance contributes to oral outcomes and quality of life at the population level. In one population-based study, dental anxiety provided little explanation for socioeconomic inequalities in most perceived and clinical oral health outcomes, although a possible effect was observed for oral health impacts on quality of life [28]. DA may be relevant not only to individual psychological distress but also to population-level differences in oral health-related quality of life, particularly through its links with dental avoidance.

The mapping model used in this review was intentionally simplified to summarise areas of consensus across heterogeneous review types. It does not capture all plausible pathways, bidirectional relations, or confounding structures. It is also possible that psychosocial consequences, including shame and reduced quality of life, may act not only as downstream outcomes but also as mediating or reinforcing processes within broader oral–general health pathways. These possibilities were not represented explicitly in the present map, which was intentionally simplified to capture the most consistently framed review-level patterns. Much of the underlying primary literature is cross-sectional, and most reviews could not make causal claims. It is also likely that DA sits within a broader network of shared risk factors for oral and systemic diseases, including diet [29], tobacco use [30], physical inactivity [31], obesity [32,33], sleep problems [34], alcohol consumption [30], and autonomic nervous function [35], which have been associated with DA, but the evidence is still limited and needs to be confirmed. Integrating DA into common risk factor frameworks is a logical next step, but it was beyond the scope of this mapping.

Future work could integrate DA into wider common risk factor frameworks to better understand how it interacts with these broader determinants. In addition, there is a paucity of theoretical development of the mechanisms through which DA might influence oral conditions and systemic disease. This gap is reflected in our mapping. The only review that integrated systemic outcomes explicitly into the main review framing presented a model linking oral health, dietary choices, and chronic disease risk, with DA positioned as an upstream driver [19]. Future work should move beyond documenting associations and develop testable models with an explicit temporal design [36]. Methodologically, new tools are available to examine the processes by which high DA might influence wider health outcomes. Intensive longitudinal assessment can help test time-lagged processes in daily life [37]. These developments may advance the field, especially if regular assessment of DA is introduced in clinical and population studies.

This review has strengths and limitations. Transparent and structured screening and classification procedures were applied, and the visual mapping approach allowed us to clearly identify neglected areas in the existing literature, including the near absence of systematic evidence on health outcomes. These features provide a structured overview that clarifies where current knowledge is concentrated and where important gaps remain.

At the same time, several limitations should be considered. This review was not prospectively registered, and no a priori protocol was published. This reflects the fact that the study was conducted as a conceptual mapping review rather than as a pre-specified effect-synthesis review; however, it limits reproducibility and makes it more difficult to distinguish fully pre-specified decisions from procedural refinements made during the review process. The search was limited to two major databases and to English-language publications, which may have resulted in the omission of relevant grey literature or reviews published in other languages. Screening and domain classification were conducted by a single reviewer, which may have increased the risk of subjective judgment. Although we used explicit binary coding rules, full-text checking, and logged reviewer overrides of AI-generated provisional coding to improve procedural transparency, these measures did not provide independent validation of the screening or coding decisions and did not substitute for independent dual review. In addition, exclusions were not retained separately by screening stage, which reduced the transparency of the record-selection workflow; the record-selection summary should therefore not be interpreted as equivalent to a standard PRISMA-style flow diagram. Finally, this was a conceptual mapping review designed to capture whether a domain was framed within a review, not to evaluate the strength of evidence or the depth of synthesis within each review type. Therefore, the same “framed” code should not be interpreted as representing an equivalent level of evidentiary integration across narrative reviews, systematic reviews, and meta-analyses.

In addition, this review was intentionally anchored to review articles explicitly framed around dental anxiety, fear, or phobia. It therefore maps the scope of the DA-framed review literature rather than the broader literature on barriers to dental care, including reviews not explicitly framed around DA. Broader access- or avoidance-focused reviews may discuss psychosocial or systemic outcomes more often, and a wider mapping of such reviews or of primary studies would be a valuable next step.

## 5. Conclusions

In conclusion, our mapping shows that the DA review literature has grown over time, but its broad conceptual framing has remained relatively stable. Reviews most commonly framed DA within a core pathway linking psychological and environmental determinants to avoidance and poorer oral health. By contrast, broader psychosocial and systemic consequences remained limited across the review literature, and systemic health outcomes were usually framed only in broad or tentative terms. Future research should therefore test hypothesised pathways from DA to broader health consequences using clearly specified bridge mechanisms and appropriate temporal designs. Such work may help place DA more clearly within a public health and equity framework.

## Figures and Tables

**Table 1:** Operational definition of framed/not framed coding.

Code	Operational Definition
Framed	Explicitly used as part of how the review defined, positioned, explained, or contextualied dental anxiety.
Not framed	Not used in that way, and present only as a peripheral mention, illustrative listing, supplementary remark, or brief future research suggestion not integrated into the review framing.

## Data Availability

The list of all screened records is publicly available in an open repository at https://doi.org/10.5281/zenodo.18523631. The full list of included reviews and the rating table, the draft mapping review checklist adapted from Li et al., the AI prompts used in this study, and the GAMER checklist are provided in the Supplementary Materials. Further inquiries can be directed to the corresponding author.

## Supplementary Materials

The following supporting information can be downloaded at: https://www.mdpi.com/article/10.3390/dj14040227/s1, File S1. The draft mapping review checklist adapted from Li et al.; File S2. AI-assisted coding support prompt used for domain classification with an unedited response; File S3. GAMER Checklist; Table S1. Included review articles and domain coding with rationale (2005–2025); Table S2. Summary of the Seven Reviews Framing Systemic Health and the Specificity of Their References to Systemic Health Outcomes. The full screening log is available via Zenodo (https://doi.org/10.5281/zenodo.18523631).

## Abbreviations

The following abbreviations are used in this manuscript:

DA	Dental anxiety
PRISMA-ScR	Preferred Reporting Items for Systematic Reviews and Meta-Analyses extension for Scoping Reviews
GAMER	Reporting guideline for the use of Generative Artificial intelligence tools in Medical Research
OHRQoL	Oral Health–Related Quality of Life
QoL	Quality of Life
WHO	World Health Organization
UHC	Universal Health Coverage
