# Supplementary material for: Dental Anxiety as a Potential Bottleneck in Oral–Systemic Health Pathways: A Conceptual Mapping Review of Review Articles
Source: Dent J (Basel). 2026 Apr 10;14(4):227. doi: 10.3390/dj14040227 (PMC13115444; doi:10.3390/dj14040227)
Supplement: Supplementary file 1 [file dentistry-14-00227-s001.zip › File S1.pdf]

File S1. The draft mapping review checklist adapted from Li et al.

Manuscript title:

Dental anxiety as a bottleneck in oral–systemic health pathways: a conceptual mapping review of review articles

Guideline:

Li, Y.; Ghogomu, E.; Hui, X.; Fenfen, E.; Campbell, F.; Khalil, H.; Li, X.; Gaarder, M.; Nduku, P.M.; White, H.; et al. Key concepts and reporting recommendations for mapping reviews: A scoping review of 68 guidance and methodological studies. *Research Synthesis Methods* **2025**, *16*, 157-174, doi:10.1017/rsm.2024.9.

Checklist

Enter the manuscript page number(s) for each applicable item.

Section	Topic	Item #	Checklist item	Page no(s)
**Title**	Title	**1**	Specify the scope and identify the report as a mapping review, evidence and gap map, or both	Yes, 1
**Authors**	Authors	**2**	List names and affiliations of all authors	Yes, 1
**Abstract**	Structured summary	**3**	Provide a structured summary that includes (as applicable) background, including the rationale and objective for the review*; methods, including stakeholder engagement, protocol, eligibility criteria, search, coding, critical appraisal, and data presentation and analyses (specifically mention the strategy for adequacy and priority setting); results, including study selection, characteristics, risk of bias, and mapping analyses; conclusions, including a summary of the main findings and implications of those findings	Partly, 1
**Background**	Rationale	**4**	Describe the scope of the review and explain why conducting this review is important	Yes, 1
**Background**	Objectives	**5**	Provide a structured statement of the research question(s) within a key element framework, and specify whether this review is intended for decision-making, to delineate evidence gaps and clusters for future research, or both. Utilize frameworks such as PI/ECOS (Population, Intervention/Exposure, Comparator, Outcome, and Study Design), PCC (Population, Concept, and Context), PIT (Population, Index Test, Target Condition), and others like PECO, PEO, and PO for various research questions.	Yes, 2
**Methods**	Stakeholders: identification and definition of stakeholders	**6a**	Specify the identification process of stakeholders and define the various types of stakeholders involved, such as direct users of research outputs (e.g., researchers and policy decision-makers), and those directly affected by decisions (e.g., patients in the field of medicine)	N/A
**Methods**	Stakeholder engagement	**6b**	Provide detailed descriptions of stakeholder engagement at each stage of the review process	N/A
**Methods**	Registration information	**7a**	Provide registration information for the review, or state if the review was not registered	Yes, 3

Section	Topic	Item #	Checklist item	Page no(s)
**Methods**	Reference protocol	**7b**	Apply the review protocol, or specify if no protocol was established	Yes, 3
**Methods**	Deviations from protocol	**7c**	Describe and explain any amendments made to the information provided at registration or in the protocol	N/A
**Methods**	Eligibility criteria	**8**	Specify the inclusion and exclusion criteria for the review, defining characteristics of the study within a key element framework based on different research questions.	Yes, 3
**Methods**	Search sources	**9**	Present all databases, registers, websites, organizations, reference lists, and other sources searched or consulted to identify studies, along with the dates when each source was last searched or consulted. If machine learning was utilized for literature search, present the details of the sources from which the literature was drawn	Yes, 3
**Methods**	Search strategy	**10**	Present the full search strategies for all search sources, including any filters and limits used. If machine learning was utilized for literature search, present the details of the relevant software and its search strings	Yes, 4
**Methods**	Screening process	**11**	Provide the process for literature screening, including at the title/abstract and full texts levels, and clarify the methods ensuring the repeatability of this process, such as specifying the number of people involved and whether they worked independently. If machine learning was used for literature screening, specify the details of how inclusion decisions were made	Partly, 4
**Methods**	Data items	**12a**	List all variables used for data extraction and/or further coding	Yes, 5
**Methods**	Development of coding tools	**12b**	Define and provide details on the analytic or consensus-based conceptual framework developed for developing coding tools	Yes, 5
**Methods**	Data collection process	**13**	Provide the process for data extraction from reports of included studies and coding, and clarify the methods ensuring the repeatability of this process, such as specifying the number of people involved and whether they worked independently. If machine learning was used for data extraction, specify the details of the relevant software and the logic used for extracting key fields.	Yes, 4
**Methods**	Critical appraisal	**14**	Specify whether the quality of included studies or reviews was assessed. If done, describe the methods used for assessment and the procedures ensuring the repeatability of the assessment process, such as specifying the number of people involved and whether they worked independently.	N/A
**Methods**	Types of presentation	**15a**	Specify the presentation formats for the mapping results of included studies, such as charts, tables, and interactive maps	Yes, 5
**Methods**	Tools for mapping	**15b**	Describe the tools or software details used for generating mapping results, including any automation or artificial intelligence software	Yes, 6
**Methods**	Dimensions in maps	**15c**	Specify the dimensions or coordinate matrix and filters (if possible) used for positioning within the maps. If Evidence Gap Maps (EGMs) tools are utilized, consult the PRISMA-EGM guidelines as necessary	Yes, 5
**Methods**	Strategy for adequacy and priority setting	**15d**	Specify strategy for determining areas with sufficient evidence, aimed at supporting decision-making and future research priorities, including evidence gaps and clusters. If possible, specify the method for ranking these priorities	Yes, 6
**Methods**	Data analysis methods	**15e**	Describe the methods used in the data analysis process, such as descriptive analysis, thematic analysis, and statistical analysis, among others	Yes, 5
**Results**	Study selection	**16a**	Describe the results of the search and selection process, from the number of references initially identified to the number of studies ultimately included in the review. Provide a flow diagram to illustrate this process	Yes, 6

Section	Topic	Item #	Checklist item	Page no(s)
**Results**	Excluded studies	**16b**	List key excluded studies that readers might reasonably expect to find and provide justification for each exclusion	No
**Results**	Study characteristics	**17**	Describe the basic study characteristics of interest. Consider equity and provide the citations, native format of extracted data and its corresponding coding for each included study, if possible.	Yes, 6
**Results**	Quality assessments	**18**	If quality was assessed, describe the quality assessments for included studies or reviews.	N/A
**Results**	Maps of included studies	**19a**	Present a map here, showing how the relevant literature is organized according to transparent, replicable key elements framework (research question), with a concise description. If possible, consider equity and include filters that allow for the generation of customized maps.	Yes, 7
**Results**	Areas with adequate evidence	**19b**	Provide a structured report within a key element framework detailing areas with sufficient evidence support for decision-making based on the strategy for adequacy, if possible	N/A
**Results**	Evidence gaps and clusters	**19c**	Provide a structured report within a key element framework detailing areas requiring further research, including primary studies and additional evidence synthesis, based on the strategy for priority setting, if possible	Yes, 7–8
**Discussion**	Summary of main results	**20**	Describe the main findings and provide a general interpretation of the results within the context of other evidence	Yes, 8
**Discussion**	Limitations of the review	**21**	Discuss the limitations of both the review processes and the included evidence	Yes, 10–11
**Discussion**	Implications	**22**	Discuss the implications for decision-making and future research in a structured manner based on key elements framework, if possible	Yes, 11
**Discussion**	Plans for map updates	**23**	Discuss the necessity of updating the map based on current research results and trends, and provide details of the update plan, including timing and content to be updated.	No
**Conclusions**	Conclusions	**24**	Report the main findings of the review and summarize the implications of those findings	Yes, 11
**Acknowledgements**	Acknowledgements	**25**	Acknowledge the contributions of individuals who are not listed as authors of the review	N/A
**Contributions of authors**	Contributions of authors	**26**	Detail the specific contributions of each author	Yes, 11
**Declarations of interest**	Declarations of interest	**27**	Report any competing interests of the review authors	Yes, 11
**Sources of support**	Sources of support	**28**	Describe the sources of financial or non-financial support for the review and specify the role of the funder	Yes, 11
