# Supplementary material for: Dental Anxiety as a Potential Bottleneck in Oral–Systemic Health Pathways: A Conceptual Mapping Review of Review Articles
Source: Dent J (Basel). 2026 Apr 10;14(4):227. doi: 10.3390/dj14040227 (PMC13115444; doi:10.3390/dj14040227)
Supplement: Supplementary file 1 [file dentistry-14-00227-s001.zip › File S2.pdf]

File S2. AI-assisted coding support prompt used for domain classification with an unedited response

Prompt:

You are assisting with a conceptual mapping review of review articles on dental anxiety (DA).

TASK

For the provided review article (PDF), classify it into the domains below and produce a short Evidence_note and Location(s).

This is not a causal adjudication and not an assessment of empirical strength.

Classify domains based on whether they are framed within the review, that is, whether they are explicitly used to define, position, explain, or contextualize dental anxiety.

COLUMNS (output one row)

Gene | Psychological | Physiological | SocialEnv | Avoidance | OralOutcomes | Shame | OHRQoL | SystemicOutcomes | QoL_General | Evidence_note | Location

CODING SCHEME

Use:

- 1 = framed
- 0 = not framed

DEFINITIONS

Code 1 = Framed

The domain was explicitly used in the review to define, position, explain, or contextualize dental anxiety.

This included domains treated as:

- the main topic of the review, or
- part of the review's background assumptions, introduction, definitional framing, conceptual explanation, or overall narrative flow

A domain may therefore be coded 1 even if it is not the main synthesis target, provided that it is clearly used to locate or explain dental anxiety within the review.

Examples of Framed:

- A review focuses on genetic factors and explains DA through inherited vulnerability (Gene = framed)
- The introduction positions DA as an important problem because it leads to avoidance of dental care and poorer oral health (Avoidance, OralOutcomes = framed)
- The background describes DA as relevant to quality of life (OHRQoL or QoL_General = framed)
- An environmental review explains DA through family factors, prior dental experiences, or treatment setting (SocialEnv = framed)

Code 0 = Not framed

The domain was not used to define, position, explain, or contextualize dental anxiety, and appeared only as:

- a supplementary remark

• a peripheral mention

• an illustrative listing

• a future research direction

Examples of Not framed:

• Mentioned once at the end as a future research need

• Referred to only in passing without being used in the review's background, explanation, or narrative framing

IMPORTANT CONCEPTUAL RULE

This mapping is intended to capture review-level framing, not whether a domain was formally tested, systematically synthesized, or empirically confirmed within that review.

Therefore:

• A domain can be coded 1 even if it appears mainly in the background, introduction, or definitional framing, as long as it is clearly part of how the review positions or explains DA.

• Do not require statistically significant findings, meta-analytic support, or detailed result synthesis for a 1 code.

• If a domain is only named or briefly noted without a clear role in framing DA, use 0.

DOMAIN DEFINITIONS

Upstream / determinant domains

• Gene: genetic factors, heritability, inherited vulnerability

• Psychological: cognition, anxiety/fear traits, temperament, personality, beliefs, mental health symptoms, emotional vulnerability

• Physiological: sensory sensitivity, pain sensitivity, autonomic reactivity, hormonal/biological processes, bodily vulnerability

• SocialEnv: family, parenting, learning history, prior dental experiences, trauma, clinic factors, dentist factors, SES, media/information, cultural context, broader environment

Downstream / consequence domains

Use these when the review frames the domain as part of the downstream significance, consequences, or broader conceptual extension of DA:

• Avoidance: avoidance of care, delayed attendance, irregular attendance, return reluctance, utilization barriers

• OralOutcomes: poorer oral health, oral disease, caries, periodontal problems, tooth loss, oral deterioration, treatment-related oral consequences if framed as outcomes or significance of DA

• Shame: shame, embarrassment, stigma, humiliation

• OHRQoL: oral health-related quality of life, oral impacts on daily life

• SystemicOutcomes: systemic disease, general health consequences beyond the mouth

• QoL_General: general quality of life, broader well-being, or daily life impairment not specifically oral-health-related

IMPORTANT RULE FOR DIRECTION

Direction still matters, but framing is the priority.

- If a downstream domain is used to show why DA matters, what DA may lead to, or how DA is positioned in a broader pathway, code it 1.
- If the same construct is discussed only as an antecedent of DA and not as part of the downstream framing, do not code it in the downstream column.
- If a bidirectional or cyclical relationship is described, downstream columns may be coded when DA is clearly positioned as contributing to that pathway.

CLARIFICATION FOR PAIN

- Code procedure-related painful dental experiences (e.g., prior painful treatment, drilling without anesthesia, traumatic visits) under SocialEnv when framed as antecedent experiences contributing to DA.
- Code pain sensitivity, sensory sensitivity, or other stable bodily vulnerabilities under Physiological.
- If pain during dental treatment is framed as an outcome, consequence, or broader significance of DA, it may be coded under OralOutcomes.

EVIDENCE_NOTE REQUIREMENTS

- English, 1–2 sentences, max 35 words
- Summarize how the review frames the main relevant domains
- Prioritize the review's main conceptual logic
- If relevant, briefly note antecedent-only or bidirectional framing
- For borderline cases, explicitly note the ambiguity using brief wording such as:
  - "borderline framing"
  - "weakly framed"
  - "framed mainly in background"
  - "only weakly integrated into framing"
- Avoid long quotations; paraphrase preferred

LOCATION REQUIREMENTS

- Provide the section(s) where the framing is supported
- Use section labels such as: Abstract, Introduction, Methods, Results, Discussion, Conclusion
- If available, add section heading names in parentheses
- Do not invent headings
- Location does not need to be exhaustive; provide the main supporting locations

BORDERLINE CASE RULE

If uncertain, ask:

Is this domain clearly used as part of how the review defines, positions, explains, or contextualizes dental anxiety?

- If yes, use 1
- If no, use 0

If the case is genuinely borderline, still assign 1 or 0, but explicitly note the ambiguity in the Evidence_note. When still uncertain, lean conservatively toward 0.

IMPORTANT

- Do not guess if information is insufficient.
- Base coding on the full review framing, not only title or abstract.
- Do not treat “framed” as equivalent to “empirically proven.”
- The goal is to map how far dental anxiety is conceptually extended across domains in the review literature, not which domains were most rigorously tested.

Raw response:

Gene	Psycho-logical	Physio-logical	Social Env	Avoidance	Oral Outcomes	Shame	OHR QoL	Systemic Outcomes	QoL_General	Evidence_note	Location
0	1	1	1	1	1	0	1	1	1	DA is framed through cognitive/constitutional and family/cultural etiology, and through avoidance leading to oral-health and QoL consequences; systemic outcomes are weakly framed as broader implications of poor dental health.	Abstract; Introduction; 4. Clinical significance; 5.2–5.5 Etiology and maintenance (*The role of cognitive and constitutional factors*; *The role of family and cultural factors*; *The role of learning, cognition, and culture in the maintenance of dental anxiety*); 6.2 Self-reports
