# Supplementary material for: Dental Anxiety as a Potential Bottleneck in Oral–Systemic Health Pathways: A Conceptual Mapping Review of Review Articles
Source: Dent J (Basel). 2026 Apr 10;14(4):227. doi: 10.3390/dj14040227 (PMC13115444; doi:10.3390/dj14040227)
Supplement: Supplementary file 1 [file dentistry-14-00227-s001.zip › File S3. GAMER_checklist_table.pdf]

Manuscript title:

Dental anxiety as a bottleneck in oral–systemic health pathways: a conceptual mapping review of review articles

Guideline:

Luo X, Tham YC, Giuffrè M, Ranisch R, Daher M, Lam K, Eriksen AV, Hsu CW, Ozaki A, Moraes FY, Khanna S, Su KP, Begagić E, Bian Z, Chen Y, Estill J; GAMER Working Group. Reporting guideline for the use of Generative Artificial intelligence tools in MEdical Research: the GAMER Statement. BMJ Evid Based Med. 2025;30(6):390–400. doi:10.1136/bmjebm-2025-113825.

GAMER checklist

No.	Item	Reported	Page
1	Did you use any GAI tools (such as large language models or large visual models) in any section or step of this manuscript or study?	■Yes □No □N/A	
2	Specify the GAI tool(s) used, their versions and/or release dates and the date(s)/period the tools were used.	■Yes □No □N/A	5
3	Describe whether a specific prompting technique was used to generate any content of the manuscript or to perform analyses during the study. Please also provide the unedited responses to the prompts.	■Yes □No □N/A	5
4	If a new GAI tool was developed or fine-tuned based on an existing AI model, report the name and version of the original model.	□Yes □No ■N/A	
5	Describe the role of GAI tools in all phases of this study where they were used (including manuscript writing).	■Yes □No □N/A	11
6	Report the specific section or paragraphs of the manuscript that GAI tools contributed to.	■Yes □No □N/A	5
7	Describe how the content generated by GAI tools was verified and (when necessary) modified.	■Yes □No □N/A	5

8	Describe how data privacy and confidentiality were ensured during the use of GAI tools.	■Yes □No □N/A	5
9	Describe whether and how the use of GAI tools may have influenced the interpretation of results, the study's overall accuracy, or conclusions.	■Yes □No □N/A	5

AI, artificial intelligence; GAI, generative artificial intelligence; GAMER, Generative Artificial intelligence tools in MEdical Research; N/A, not applicable.
