# Supplementary material for: Dental Anxiety as a Potential Bottleneck in Oral–Systemic Health Pathways: A Conceptual Mapping Review of Review Articles
Source: Dent J (Basel). 2026 Apr 10;14(4):227. doi: 10.3390/dj14040227 (PMC13115444; doi:10.3390/dj14040227)
Supplement: Supplementary file 1 [file dentistry-14-00227-s001.zip › Table S2.pdf]

Supplementary Table S2. Summary of the Seven Reviews Framing Systemic Health and the Specificity of Their References to Systemic Health Outcomes

Author	Year	Review type	Population focus	Main focus	How systemic health was framed	Specificity
Slovin & Falagario-Wasserman	2009	Narrative / clinical review	General / mainly adult clinical context	Management and special needs of anxious and phobic dental patients	In the Introduction, systemic health was mentioned broadly, with oral problems related to dental avoidance described as potentially affecting systemic and overall health.	**Low** – broad systemic-health wording only, without any named disease example.
Seligman et al.	2017	Narrative review	Child / youth	Dental anxiety in youth	In clinical significance / introductory framing, broader health implications were noted, including links to systemic inflammation, cardiovascular disease risk, and obesity. In the Conclusions/Future directions, the review stated that direct pathways from dental anxiety to later health complications had not yet been established.	**Moderate** – named disease examples and a tentative broader-health pathway were mentioned, but this remained secondary and cautious rather than centrally synthesised.
Beaudette et al.	2017	Narrative review	Adult / general dental patients	Oral health, nutritional choices, and dental fear/anxiety	In the Abstract and Introduction, poor oral health was linked to chronic disease risk. In Figure 1, an indirect pathway was explicitly presented: fear/anxiety → treatment avoidance → compromised dentition / limited food choice → chronic disease risk. In the Discussion/Conclusion, this broader oral–nutrition–general health link was reinforced.	**High** – explicit indirect mechanism and multiple named systemic diseases.
Tarrosh et al.	2022	Systematic review	General (with child/adolescent developmental framing in background)	Dental anxiety levels across genders and demographic groups in Saudi Arabia	In the Background/Introduction, poor oral health related to dental anxiety was described as undermining growth and development and predisposing children/adolescents to systemic disease. In the Discussion, only broad health-burden language appeared.	**Low** – systemic disease was mentioned only broadly as a downstream consequence, without any named disease example.

Ying et al.	2023	Narrative review	Child	Children’s dental fear: occurrence mechanisms and prevention	In sections on pre-existing illnesses and hazards of Children’s dental fear, broader health consequences were discussed, including growth/development effects, malnutrition, and later health complications. In the discussion of hazards/future outlook, cardiovascular disease and obesity were given as examples, while noting that direct evidence remained unestablished.	**Moderate** – named disease examples and concrete downstream consequences were mentioned, but these remained peripheral to the main review focus.
Farias et al.	2023	Systematic review	General / mainly endodontic patients	Anxiety, pain perception, and repercussions for endodontic treatment	In the Abstract/clinical relevance and Introduction, anxiety was linked to treatment delay/evasion and worsening infection. In the Discussion, this was extended to worse general health.	**Low** – infection-related and general-health consequences were mentioned, but without any named systemic disease example.
Aburas et al.	2023	Umbrella review	General	Dentophobia and dental treatment	In the main text discussion of emotional/behavioural symptoms, worsening general health and endocarditis were mentioned as downstream consequences of avoidance and poor oral health.	**Moderate** – one named disease example was provided, but systemic-health framing was otherwise broad and peripheral.

Specificity was classified as low when systemic health was mentioned only in broad terms; moderate when at least one named disease example and/or a tentative downstream pathway was provided; and high when an explicit indirect mechanism and multiple named systemic diseases were integrated into the main review framing.
